# Acute effects of tissue flossing on boxers with chronic knee pain

**DOI:** 10.3389/fbioe.2024.1508054

**Published:** 2025-01-14

**Authors:** Jie Chen, Qirong Wang, Zhiguang Zhao, Qipeng Song, Peng Zhao, Dan Wang

**Affiliations:** ^1^ School of Athletic Performance, Shanghai University of Sport, Shanghai, China; ^2^ Sports Nutrition Center, National Institute of Sports Medicine, Beijing, China; ^3^ College of Sports and Health, Shandong Sport University, Jinan, China; ^4^ Sports Science Research Institute of the State Sports General Administration, Beijing, China

**Keywords:** voodoo flossband, blood flow restriction, knee joint, pain, muscle strength

## Abstract

**Objectives:**

To explore the acute intervention effects of tissue flossing on chronic knee pain (CKP) in boxers.

**Methods:**

Eighteen boxers with CKP (12 male/6 female) were randomly divided into an experimental group (EG) with tissue flossing (n = 9) and a control group (CG) (n = 9). The visual analog scale (VAS), Lysholm knee function score, flexion range of motion (ROM), maximal isometric extensor muscle strength, and stability of the knee were measured pre- and post-intervention (EG: 3-minute tissue flossing, CG: rest).

**Results:**

VAS (*F* = 15.849, *p* = 0.001, *η* ^*2*^_*p*_ = 0.498) and Lysholm knee function (*F* = 9.327, *p* = 0.008, *η* ^*2*^_*p*_ = 0.357) significantly improved more in the EG than in the CG. There was a significant difference for maximal isometric extensor muscle strength (*F* = 17.769, *p* = 0.001, *η* ^*2*^_*p*_ = 0.542) and knee stability (*F* = 13.844, *p* = 0.002, *η* ^*2*^_*p*_ = 0.464) but no significant difference for ROM (*F* = 1.218, *p* = 0.287, *η* ^*2*^_*p*_ = 0.075) between the EG and CG.

**Conclusion:**

Tissue flossing can reduce knee pain, improve knee function, increase knee extensor strength, and improve knee stability in boxers with CKP.

## 1 Introduction

Boxing is an activity prone to injury. During boxing competitions, the incidence rate of lower extremity injuries among boxers is 1,220 per 1,000 h (Zhou et al., 2022). Boxers may also suffer such injuries due to overuse, amounting to 21.6% of total injuries (Zhou et al., 2022). A study by the British boxing team showed that knee injuries (any musculoskeletal condition that prevented the boxer from participating in either training or competition for >24 h) accounted for 25% of all injuries in boxers (Loosemore et al., 2015). Therefore, knee injuries are quite common in boxers and thus require attention from researchers.

The main factor associated with knee injuries in boxers is incorrect knee movement patterns (Wilczyński et al., 2020), including technical movements specific to boxing and incorrect squat techniques (Duong et al., 2023). For example, jabs and crosses are two scoring techniques that require a boxer’s knee to be in an internal rotation position, adding an additional load to the knee. Repeated practice in this specific position leads to an increased risk of injury to the knee joint (Duong et al., 2023). Specifically, the knee abduction moment increases with greater knee internal rotation (Wang et al., 2022). An elevated knee abduction moment is associated with a higher risk of anterior cruciate ligament injury (Ekdahl et al., 2024) and increased tibiofemoral cartilage contact pressure (Erbulut et al., 2021), which can contribute to the development of overuse injuries such as early osteoarthritis (Pache et al., 2018). Squats are commonly employed to enhance lower extremity strength in boxers (Escamilla, 2001), which can increase the power of their punches through kinetic chains (Zhou et al., 2022). A correlation between the maximal isometric strength in half-squat position and the effectiveness of jabs (r = 0.68) and the crosses (r = 0.83) was reported in boxing (Zhou et al., 2022). However, athletes often exhibit incorrect techniques when performing squats, such as the knee in an internal rotation position or knee-dominated squats, which increase the risk of knee injuries (Duong et al., 2023). The average recovery time that can attend training after knee damage in boxers was reported to be 21 days (Loosemore et al., 2015). Due to the continuous and long-term nature of training, athletes frequently do not receive adequate rest and treatment following injuries, leading to the development of chronic knee pain (CKP).

CKP refers to knee pain lasting more than 3 months (Zhang et al., 2017). This pain is primarily a manifestation of damage to the structures within the knee, usually accompanied by decreases in the level of knee function (Ventura et al., 2018), such as limited range of motion (ROM), reduced muscle strength around the knee, and decreased joint stability. Tissue flossing—otherwise known as voodoo band or floss band—is a natural rubber tape strip with a certain thickness and excellent elasticity (Wu et al., 2022). It features low allergic reactions and ultra-strong elasticity characteristics. Currently, it is commonly used in the field of rehabilitation and is considered a novel tool for treatment (Driller and Overmayer, 2017b; Driller et al., 2017a; Marco A. et al., 2020; Wienke et al., 2020; Wu et al., 2022). Tissue flossing applies pressure to joints and muscles, which enhances joint function (e.g., ROM) (Wu et al., 2022), improves muscle function (e.g., muscle strength) (Kaneda et al., 2019), and promotes recovery (e.g., relieving knee pain) (Marco A. et al., 2020). The only existing study on the therapeutic effects of tissue flossing found that an acute intervention significantly improved knee pain in individuals with patellofemoral pain syndrome (Marco A. et al., 2020). However, this was a pilot study involving only five amateur athletes and utilized a crossover study design (Marco A. et al., 2020). The effect produced by tissue flossing can last at least 20 min (Wu et al., 2022). For boxers engaged in 3-round, 3-minute competitions with 1-minute breaks in between, totaling 11 min, tissue flossing might help alleviate pain during competition or training and potentially enhance performance. Therefore, we decided to implement tissue flossing for boxers with CKP. The main mechanisms include fascial shear (Stecco et al., 2013), pain gate control (Moayedi and Davis, 2013), and blood flow restriction and reperfusion (Reeves et al., 2006; Hughes et al., 2017). The pressure exerted by tissue flossing on joints and muscles is in the form of shear forces (Stecco et al., 2013), which can loosen adhesion in muscle tissues and reduce the viscosity of hyaluronic acid to restore lubricating effects, thereby increasing joint mobility (Stecco et al., 2013). It also stimulates large myelinated nerve fibers (type Ⅰ afferent neurons), which generate nerve impulses that inhibit the transmission of pain signals, thereby reducing the sensation of pain (Moayedi and Davis, 2013). Simultaneously, the pressure generated by the tissue flossing restricts the blood flow to the joint, muscles, and tissue, causing muscles to temporarily enter an anaerobic state and altering the metabolic type of the muscle (Reeves et al., 2006; Hughes et al., 2017). Upon the removal of the fascial compression band, there is a substantial influx of blood, which creates a reperfusion effect. This enhances vasodilation, eliminates muscle tissue waste, and nourishes the muscles, thereby improving muscle contraction efficiency (Reeves et al., 2006; Hughes et al., 2017).

At present, few studies have explored the effect of tissue flossing at different pressure values on warm-up or treatment. Galis et al. applied 150 mmHg as the low pressure and 200 mmHg as the high pressure during a tissue flossing intervention, finding significant improvement in ankle dorsiflexion only at the lower pressure (Galis and Cooper, 2022). Therefore, controlling the pressure value of the tissue flossing at 150 mmHg may produce a positive treatment effect. The recommended duration for tissue flossing is 1–3 min (Konrad et al., 2021). One-minute tissue flossing has been proven to improve countermovement jump and relieve pain (Marco A. et al., 2020). However, this research was conducted among amateur athletes (Marco A. et al., 2020), and a 1-minute intervention may not yield the same effect on professional athletes. Two minutes of tissue flossing have been shown to significantly improve hamstring flexibility without notably enhancing jump power (Maust et al., 2021). In contrast, 3 minutes of tissue flossing not only improves hamstring flexibility, but also enhances landing stabilization (Wu et al., 2022). More importantly, this study found that the effects (improved hamstring flexibility and landing stabilization) of a 3-minute tissue flossing last for at least 20 min (Wu et al., 2022), well exceeding the 11-minute duration of a boxing competition. Therefore, a 3- minute tissue flossing intervention was selected for the present study.

As a rehabilitation approach to improve CKP symptoms, the tissue flossing may provide an efficient therapeutic method for boxers with CKP, as well as other athletes who engaged in lower-extremity-demanding sports. Currently, there has been no research examining the immediate effects of applying tissue flossing in boxers with CKP. Therefore, this study aims to investigate the acute effects of tissue flossing on boxers suffering from CKP. It is hypothesized that immediately following a 3-minute tissue flossing intervention, boxers with CKP can exhibit significant improvements in knee pain, function, ROM, muscle strength, and stability.

## 2 Materials and methods

### 2.1 Participants

#### 2.1.1 Sample size calculation

G*Power was designed as a general stand-alone power analysis program for statistical tests commonly used in social and behavioral research (Faul et al., 2007). It provides improved effect size calculators and graphic options, supports both distribution-based and design-based input modes, and offers all types of power analyses (Faul et al., 2007). G*Power 3.1 was utilized for the sample size calculation with a repeated measures ANOVA (within–between interaction). The primary outcome VAS score (the mean ± standard deviation of the VAS score: pre-intervention for experimental group (EG) and control group (CG) - 3.4 ± 1.3 vs. 4.5 ± 2.6; post-intervention for EG, and CG- 2.1 ± 0.8 vs. 4.6 ± 3.0) in the pilot study was utilized for the effect size calculation in SPSS 26.0 (IBM, United States) with two-way repeated-measures ANOVAs, and the result showed that the effect size *η* ^*2*^_*p*_ was 0.435. A minimal sample size of seven participants per group was calculated based on this effect size and a power of 0.8. Considering a 20% attrition rate (Regnaux et al., 2015; Ventura et al., 2018), nine participants for each group were required.

#### 2.1.2 Randomization

Participant recruitment was completed by the enrollment of 18 boxers with CKP from provincial boxing teams. Participants were assigned numbers: female boxers drew numbers 1 to 6, while male boxers drew numbers 7 to 18. Odd-numbered participants were assigned to the tissue flossing group (EG; n = 9), and even-numbered participants were assigned to the control group (CG; n = 9). The intervention and testing were conducted separated for each participant to ensure single blinding. The demographic information of the participants is shown in Table 1.

**TABLE 1:** Demographic information of the participants.

Gender	EG (*n* = 9)	CG (*n* = 9)
	Male (*n* = 6)/female (*n* = 3)	Male (*n* = 6)/female (*n* = 3)
Age (years)	18.9 ± 1.5	20.7 ± 2.7
Height (cm)	178.6 ± 12.1	175.7 ± 5.5
Weight (kg)	71.5 ± 21.0	67.7 ± 10.8

EG, experimental group; CG, control group.

#### 2.1.3 Inclusion and exclusion criteria

The inclusion criteria of the participants were as follows: (1) CKP patients: a visual analog scale (VAS) score of 3 points or greater experienced during training in the past month, and knee pain that has persisted for more than 3 months (Hott et al., 2015; Zhang et al., 2017; Ventura et al., 2018); (2) active professional boxers of grade II (based on the Chinese National Athletes Grading System) or above, regardless of gender; (3) aged between 18 and 24 years; and (4) no intake of medications affecting muscle performance (e.g., muscle relaxants) within the past 3 months. Inclusion criteria were designed to limit the participants to a typical boxing population in China who often suffer CKP.

Participants who (1) had experienced new injuries within the past 3 months; (2) had a history of ligament rupture, fracture, or surgery related to the knee joint or who had prosthetic devices implanted or replaced; (3) had chronic diseases may impact overall health and quality of life, such as peripheral joint disease or heart disease; and (4) had latex allergy were excluded. The exclusion criteria were to further eliminate patients with injuries other than CKP or conditions deemed unsuitable for this study, ensuring data reliability and participants safety throughout the study.

All participants signed an informed consent form. The ethical committee of the Shanghai University of Sport approved the study prior to the initiation of testing (102772024RT045). This study was conducted in accordance with the principles of the Declaration of Helsinki.

### 2.2 Intervention

A blue, medium-weight version of a 2 m × 5 cm floss band (Sanctband, Perak Darul Ridzuan, Malaysia) was used for the intervention. The pressure value of the floss band was set at 150 mmHg ± 10 mmHg (Galis and Cooper, 2022), measured using a Kikuhime pressure monitoring device (HPM-KH-01, Horsens, Denmark) (Driller et al., 2017a). The device has proven to have high reliability (ICC = 0.99, CV = 1.1%) and validity (CV = 4.9%) (Mills et al., 2020).

Each participant in the EG stood with their affected knee slightly bent, and a pressure monitor was placed on the lateral condyle of the femur (Marco A. et al., 2020). The floss band was then wrapped from their tibial tuberosity upward to 5 cm above the patella on the femur, ensuring that the patella was exposed when passing over. The first wrap was applied without tension for fixation, while subsequent wraps required 50% tension, with each successive wrap overlapping the previous one by half to ensure continuity between the layers of the floss band. Participants in the EG sat quietly for 3 min with tissue flossing (Wu et al., 2022), while the participants in the CG sat quietly for 3 min without any intervention.

### 2.3 Measurements

**FIGURE 1:**
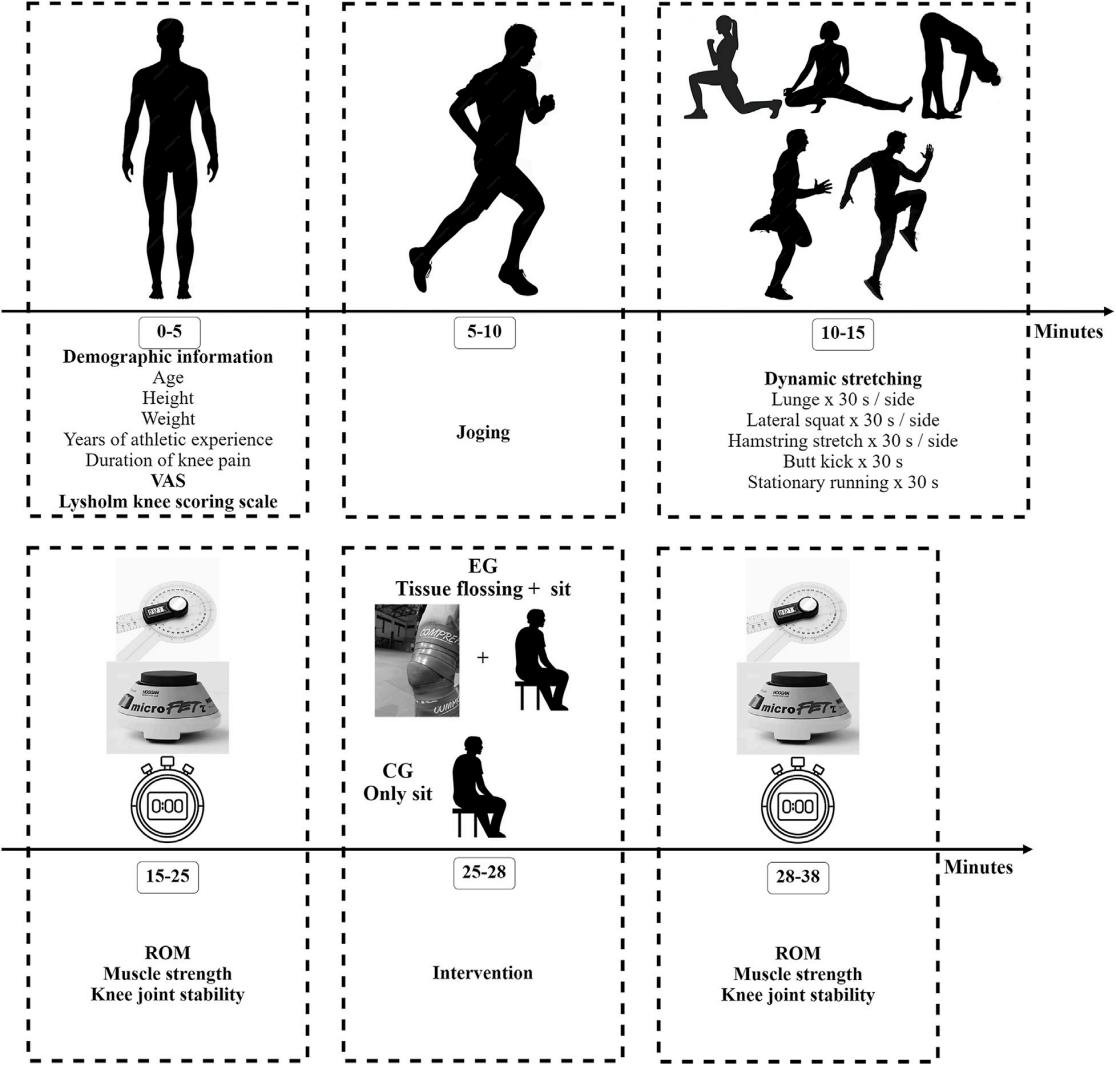
Study flowchart VAS, visual analogue scale; ROM, range of motion; EG, experiment group; CG, control group.

The VAS score of knee pain and the Lysholm knee scoring scale were measured first. Afterwards, all the participants engaged in a 5-minute jog, followed by another 5 min of dynamic stretching (DeVita et al., 2018) for warm-up. The dynamic stretching routine included lunges (15 repetitions, 2 s per repetition, 30s/side, total 1min), lateral squats (15 repetitions, 2 s per repetition, 30 s/side, total 1 min), hamstring stretches (15 repetitions, 2 s per repetition, 30 s/side, total 1 min), butt kicks (30 s), and stationary running (30 s) (Su et al., 2017). The ROM of the knee, maximal isometric strength of the knee extensor muscles, and single-leg stance with eyes closed were evaluated (Figure 1).

### 2.4 VAS score of knee pain

The VAS was used to subjectively assess the degree of knee pain. This ranged from “no pain” to “the worst imaginable pain” at the respective ends (Heller et al., 2016). Each participant marked a vertical line on the scale, and the length from the leftmost end to the mark was measured to the nearest millimeter to represent the level of pain (Heller et al., 2016). The scale has been shown to have high reliability (ICC = 0.90) and validity (CV = 7.29%) (Paungmali et al., 2012).

### 2.5 Lysholm knee scoring scale

The Lysholm knee scoring scale was utilized to evaluate the impact of CKP on the daily activities of the participants, who filled in the questionnaire based on their experiences. The scale consists of eight items: pain, instability, locking sensation, swelling, limping, climbing stairs, squatting, and the use of walking aids. The total score ranges from 0 to 100 points, with higher scores indicating better knee function. Scores above 95 are considered excellent, 85–94 are good, 65–84 are fair, and below 65 are poor (Briggs et al., 2009; Collins et al., 2011). The scale has demonstrated high reliability (ICC = 0.935) and acceptable internal consistency (Cronbach’s alpha = 0.726) (Wang et al., 2016).

### 2.6 ROM

Each participant lay prone on the examining table. The researcher placed the center of the electronic goniometer (GemRed, Guangxi Crystal Sensor Technology Co., Ltd., Guangxi, China) on the lateral condyle of the knee joint. The fixed arm was aligned parallel to the longitudinal axis of the femur, while the moving arm was aligned parallel to the line between the lateral malleolus and the head of the fibula. Upon the researcher’s command, the participant slowly flexed their knee to its maximum, at which point the researcher recorded the angle. To avoid compensatory movements, such as hip rotation, flexion, or abduction, verbal reminders were provided throughout the measurement process. Three measurements were taken with a 30-second interval between each, and the average was utilized for further analysis (Dos Santos et al., 2017).

### 2.7 Maximal isometric strength of the knee extensor muscles

The researcher used a hand-held dynamometer (Micro FET2, Hoggan Health Industries, Draper, USA) to measure the maximal isometric strength of the knee extensor muscles (Stark et al., 2011). The reliability of this device in strength measurement has proven to be high (ICC = 0.90) (Mentiplay et al., 2015).

Each participant sat at the edge of an examining table with both legs dangling freely, and it was ensured that the hips and knees were positioned at a 90° angle. They were required to maintain an upright torso and hold onto the edge of the examining table with both hands. The researcher stood against a wall, aligning their back with it, and placed the hand-held dynamometer on the front part of the participant’s shin near the proximal ankle joint. Upon the researcher’s command, the participant exerted maximum force against the dynamometer for 5 s. This measurement was repeated three times, with 30 s between each measurement (Martin et al., 2006). The highest value was recorded for further analysis. Throughout the testing process, the researcher provided ongoing verbal encouragement to ensure that the participants continuously maintained maximum effort (Martin et al., 2006).

### 2.8 Single-leg stance with eyes closed

Knee pain can change proprioceptive information (Bennell et al., 2008) and reduce proprioception (Kavchak et al., 2012), and thus impair knee stability (Bennell et al., 2008). The single-leg stance with eyes closed can reflect knee stability and proprioception without visual input (Baumann et al., 2017; Wu et al., 2022). Therefore, it was used as the measurement of knee joint stability. Each participant stood naturally with their eyes closed. Upon the researcher’s command, they lifted the unaffected leg and started being timed for the duration of their stance. The timer stopped when the supporting foot moved or the lifting foot touched the ground. The measurement was performed twice, and the better result was utilized for further analysis.

### 2.9 Statistical analysis

Data analysis was conducted using SPSS 26.0 (IBM, United States). The normality of the data distribution was tested using the Shapiro–Wilk test. For data meeting the normal distribution, means ± standard deviations (*M* ± *SD*) were used to present the data, while median and interquartile range *M* (*P*_*25*_, *P*_*75*_) were used for data that did not follow a normal distribution. The baselines were analyzed using an independent-samples *t*-test. If there were no significant differences at the baseline, two-way repeated-measures ANOVAs were utilized for the data analysis, which considered time (pre-intervention vs. post-intervention) and group (EG vs. CG) as factors affecting related variables (knee VAS score, Lysholm knee function score, ROM of knee, maximum isometric muscle strength of the knee extensors, and single-leg stance time with eyes closed). If there was an interaction effect between time and group, a simple effects analysis was performed. If data at the baseline were significantly different, the data were analyzed using an analysis of covariance to compare differences between the EG and CG (Hara et al., 2020). The aligned rank transform (ART) was used by ARTool 2.2.2 to transform the nonparametric data, and then two-way ANOVA was used to analyze the transformed data (Leys and Schumann, 2010; Wobbrock et al., 2011). If there was an interaction effect between time (pre-intervention vs. post-intervention) and group (EG vs. CG), a simple effects analysis was performed (Leys and Schumann, 2010; Wobbrock et al., 2011). Effect size was categorized as follows: 0 ≤ *η* ^*2*^_*p*_ < 0.05 (no effect), 0.05 ≤ *η* ^*2*^_*p*_ < 0.26 (small effect), 0.26 ≤ *η* ^*2*^_*p*_ < 0.64 (medium effect), and *η* ^*2*^_*p*_ ≥ 0.64 (large effect). Statistical significance for all data was set at a p-value of less than 0.05.

## 3 Results

All data fit normal distribution except single-leg stance with eyes closed. Apart from ROM and maximal isometric strength of the knee extensor muscle, the rest of the data at baseline were not significantly different (Table 2).

**TABLE 2:** Baselines of different indicators.

		Pre-(*M* ± *SD*)/*M* (*P*_25_, *P*_75_)	*t/Z*	*p*
Knee pain VAS score	EG	4.6 ± 1.9	0.359	0.724
	CG	4.2 ± 2.2		
Lysholm knee function score	EG	68.2 ± 14.0	−1.201	0.247
	CG	76.3 ± 14.6		
ROM (°)	EG	117.4 ± 7.6^†^	−3.451	0.003
	CG	128.7 ± 5.8		
Maximal isometric strength of the knee extensor muscle (kg)	EG	40.7 ± 12.3^†^	−3.588	0.002
	CG	57.4 ± 7.7		
Single-leg stand with eyes closed (s)	EG	15.23 (9.16, 72.99)	−1.280	0.200
	CG	30.59 (18.76, 38.77)		

†A significant difference between EG and CG, was observed (*p* < 0.05). VAS: visual analog scale; ROM: range of motion; EG: experimental group; CG: control group.

### 3.1 VAS score of knee pain

The results of the two-way repeated measures ANOVA (Table 3) indicated that there was a significant interaction between time and group for the VAS score of knee pain (*F* = 15.849, *p* = 0.001, *η* ^*2*^_*p*_ = 0.498).

**TABLE 3:** Changes in knee pain VAS scores.

		Pre-(*M* ± *SD*)	Post-(*M* ± *SD*)
Knee pain VAS score	EG	4.6 ± 1.9	2.7 ± 1.1*
	CG	4.2 ± 2.2	4.7 ± 2.5
	*F*	*p*	*η* ^*2*^_*p*_
Time main effect	6.046	0.026	0.274
Group main effect	0.773	0.392	0.046
Group × time	15.849	0.001	0.498

*A significant change between pre- and post-intervention was observed (*p* < 0.05). VAS: visual analog scale; EG, experimental group; CG, control group.

The results of the simple main effect of time (*F* = 6.046, *p* = 0.026, *η* ^*2*^_*p*_ = 0.274) showed a significant decrease in VAS score of knee pain in the EG (*F* = 20.737, *p* < 0.001, *η* ^*2*^_*p*_ = 0.564) and no significant change in the VAS score of knee pain in the CG (*F* = 1.159, *p* = 0.298, *η* ^*2*^_*p*_ = 0.068) post-intervention.

### 3.2 Lysholm knee function score

The results of the two-way repeated measures ANOVA (Table 4) indicated a significant interaction effect between time and group on the Lysholm knee function scores (*F* = 9.327, *p* = 0.008, *η* ^*2*^_*p*_ = 0.357).

**TABLE 4:** Changes in Lysholm knee function scores.

		Pre-(*M* ± *SD*)	Post-(*M* ± *SD*)
Lysholm knee function score	EG	68.2 ± 14.0	77.4 ± 10.2^*^
	CG	76.3 ± 14.6	76.2 ± 12.7
	*F*	*p*	*η* ^*2*^_*p*_
Time main effect	8.888	0.009	0.357
Group main effect	0.338	0.569	0.021
Group x time	9.327	0.008	0.368

*A significant change between pre- and post-intervention was observed (*p* < 0.05). EG, experimental group; CG, control group.

The simple main effect of time (*F* = 8.888, *p* = 0.009, *η* ^*2*^_*p*_ = 0.357) revealed that the Lysholm knee function scores significantly increased following the intervention in the EG (*F* = 18.213, *p* = 0.001, *η* ^*2*^_*p*_ = 0.532), but there was no significant change in the CG (*F* = 0.003, *p* = 0.960, *η* ^*2*^_*p*_ < 0.001).

### 3.3 ROM

The results of the analysis of covariance showed no significant differences between the EG and CG post-intervention (*F* = 1.218, *p* = 0.287, *η* ^*2*^_*p*_ = 0.075). However, the ROM of knee flexion increased by 6.32° in the EG post-intervention (Table 5).

**TABLE 5:** Changes in ROM.

		Pre-(*M* ± *SD*)	Post-(*M* ± *SD*)
ROM (°)	EG	117.4 ± 7.6	123.7 ± 6.0
	CG	128.7 ± 5.8	129.9 ± 5.3
Analysis of covariance	*F*	*p*	*η* ^*2*^_*p*_
	1.218	0.287	0.075

ROM, range of motion; EG, experimental group; CG, control group.

### 3.4 Maximal isometric strength of the knee extensor muscles

The results of the analysis of covariance showed a significant difference between the EG and CG post-intervention (*F* = 17.769, *p* = 0.001, *η* ^*2*^_*p*_ = 0.542) (Table 6).

**TABLE 6:** Changes in maximal isometric strength of the knee extensor muscles.

		Pre-(*M* ± *SD*)	Post-(*M* ± *SD*)
Maximal isometric strength of the knee extensor muscle (kg)	EG	40.7 ± 12.3	50.2 ± 14.4^†^
	CG	57.4 ± 7.7	55.8 ± 7.2
Analysis of covariance	*F*	*p*	*η* ^*2*^_*p*_
	17.769	0.001	0.542

†A significant difference between the EG and CG, was observed (*p* < 0.05). EG, experiment group; CG, control group.

### 3.5 Single-leg stance with eyes closed

The results of the two-way ANOVA after ART (Table 7) indicated that there was a significant interaction between time and group for the duration of the single-leg stance with eyes closed (*F* = 13.844, *p* = 0.002, *η* ^*2*^_*p*_ = 0.464).

**TABLE 7:** Changes in single-leg stance with eyes closed.

		Pre-(*M* ± *SD*)	Post-(*M* ± *SD*)
Single-leg stance with eyes closed (s)	EG	15.23 (9.16, 72.99)	31.40 (17.45, 78.07)
	CG	30.59 (18.76, 38.77)	25.98 (16.38, 35.44)
Two-way ART ANOVA	*F*	*p*	*η* ^*2*^_*p*_
Time main effect	2.141	0.163	0.118
Group main effect	0.116	0.738	0.007
Group x time	13.844	0.002	0.464

EG, experimental group; CG, control group.

The results of the simple main effect of time (*F* = 2.141, *p* = 0.163, *η* ^*2*^_*p*_ = 0.118) and group (*F* = 0.116, *p* = 0.738, *η* ^*2*^_*p*_ = 0.007) both showed no significant change in the duration of the single-leg stance with eyes closed.

## 4 Discussion

This study investigated the acute effects of tissue flossing on boxers with CKP. Our findings indicated that, after a 3-minute tissue flossing intervention, the VAS scores for knee pain, Lysholm knee scale scores, and the maximum isometric strength of the knee extensors in EG improved significantly compared to those in CG. However, there were no significant changes in knee ROM or single-leg stance duration with eyes closed. This indicates that tissue flossing can be used as a short term and highly effective method for relieving knee pain and increasing muscle strength for CKP boxers before competitions or training.

Immediately following the tissue flossing intervention, the boxers in the EG experienced a significant improvement in knee pain compared to those in the CG. Pain is the primary symptom of CKP (Hinman et al., 2015; Terry et al., 2022), and a reduction in knee muscle strength (Cheatham, 2020) and joint ROM is also associated with pain (Yoshizuka et al., 2022). In 2020, Marco A et al. conducted a study on five male athletes with patellofemoral pain syndrome, who performed three countermovement jumps with a 15-s interval between jumps while wearing tissue flossing (Marco A. et al., 2020). Their results indicated that the athletes had a significant reduction in knee pain post-intervention, consistent with the findings of this experiment (Marco A. et al., 2020). León-Morillas et al. showed that tissue flossing combined with physiotherapy for 8 weeks can significantly decrease anterior knee pain (León-Morillas et al., 2024). A case study indicated that 9 weeks of tissue flossing intervention could significantly improve knee pain (Weber, 2018). Although the two studies did not report on acute interventions for knee pain, they can prove that tissue flossing is beneficial for knee pain (Weber, 2018). There are several possible reasons why tissue flossing significantly improves knee pain. First, according to the gate control theory of pain, the mechanical pressure exerted by tissue flossing stimulates large myelinated nerve fibers (type I afferent neurons), and generates neural impulses (Moayedi and Davis, 2013; Wu et al., 2022). These large nerve fibers inhibit the activity of the small nerve fibers responsible for transmitting pain and thus suppress pain transmission and result in pain relief (Moayedi and Davis, 2013; Wu et al., 2022). Second, local mechanical pressure may reduce the influx of inflammatory mediators, leading to a decrease in intracellular osmotic pressure and consequently reducing the inflammatory response and sensitivity of pain receptors (Prill et al., 2019; Konrad et al., 2021).

Boxers in the EG showed a significant increase in the Lysholm knee scoring scale compared to those in the CG. One study indicated that the subjects showed a significant improvement in the Lysholm knee scoring scale after both an acute and 4 weeks of instrument-assisted soft tissue mobilization therapy combined with blood flow restriction training, consistent with the results of this experiment (Liu and Wu, 2023). Although this study did not utilize tissue flossing, the working mechanism of blood flow restriction was similar to tissue flossing. The difference between the studies was the accuracy of quantification of the pressure. The significant improvement in the Lysholm knee scoring scale due to tissue flossing may have been related to pain relief. The EG had an average increase of 9.22 points on the Lysholm knee scoring scale, which was primarily due to improvement in the following three sections: pain, squatting, and climbing stairs. This suggests that the tissue flossing intervention had an immediate positive effect on improving knee function and that its mechanism of action may be through pain relief to enhance the function scores of squats and climbing stairs.

The knee extensor muscle strength of the EG significantly improved when compared to that of the CG. Kaneda et al. found that immediately after a 2-minute tissue flossing intervention, there was a significant increase in the maximum eccentric contraction strength of the knee extensors (*p* = 0.02) (Kaneda et al., 2019). The results of this study were consistent with ours. Similarly, Marco A et al. demonstrated that a tissue flossing intervention could immediately significantly enhance the jump height, jump power, and jump force of the subjects (Marco A. et al., 2020). Although their study did not directly measure knee muscle strength, it indirectly reflected that the muscle strength and explosive power of the lower extremities significantly increased after a tissue flossing intervention through jumping. There are also studies that have presented opposite results. Paravlic et al. found that after three consecutive 2-minute tissue flossing interventions, there were significant decreases in jump height and the contractile characteristics of the vastus lateralis muscle (muscle contraction speed, maximum displacement amplitude) of the subjects (Paravlic et al., 2022). The authors believed that the reason for this phenomenon was related to the pressure, location, duration of the tissue flossing intervention, and different combined exercises (Paravlic et al., 2022). Besides pain relief, there may be three other reasons for the significant enhancement of knee muscle strength. First, the pressure exerted by tissue flossing on the joint is similar to blood flow restriction, which reduces blood flow and causes local muscles and joints to be in a state of hypoxia and ischemia (Reeves et al., 2006; Hughes et al., 2017). In this state, type II muscle fibers are recruited more quickly, thereby increasing muscle strength (Schiaffino and Reggiani, 2011). Second, after the removal of the tissue flossing, blood will be re-perfused into the muscle tissue (Hughes et al., 2017). At this moment, nitric oxide synthase is activated, leading to an increase in nitric oxide levels (Hughes et al., 2017). Nitric oxide causes vasodilation, temporarily increases blood flow, nourishes the muscles, improves muscle contraction efficiency, and ultimately leads to an increase in muscle strength (Maiorana et al., 2003). Third, pain has an inhibitory effect on muscles, causing a decrease in knee extensor muscle strength. The degree of muscle inhibition is proportional to the level of pain (Henriksen et al., 2011). Research has shown that for every 1-mm increase in pain using VAS to evaluate pain, there is an approximately 0.4% decrease in muscle strength (Henriksen et al., 2011). The results of this study indicate that after a 3-minute tissue flossing intervention, the pain in the knee joints of boxers decreased immediately. Therefore, the degree of muscle inhibition was reduced, and knee extensor muscle strength increased accordingly.

There was no significant change in knee flexion ROM between the EG and CG post-intervention. The results of studies by Wu et al. and Cheatham et al. were not consistent with those of the current study (Wu et al., 2022; Cheatham, 2020). Wu et al. found that flossing bands combined with movements (walking knee hugs, side squats, and forward lunges; each movement repeated 10 times within 3 min) could significantly increase the ROM of the knee. Cheatham et al. conducted an intervention with tissue flossing on the thighs of healthy individuals for 2-minute active movements (standing hip flexion for 30 s, seated knee extension and flexion for 30 s, and bodyweight squats for 1 min) and found that a 2-minute application of tissue flossing could immediately significantly enhance the ROM of the knee (an average improvement of 4°) (Cheatham, 2020). The results of the differences between the current study and the other two studies may mainly have been caused by the combined movements and the duration of the interventions. However, the current study demonstrated that ROM increased by 6.32° after the tissue flossing intervention. There were two possible reasons why the ROM of the knee was increased. First, it may be related to the relief of pain (Yoshizuka et al., 2022). Patients often adopt a strategy of immobilization to recover when they experience knee pain, and prolonged immobilization can lead to tissue adhesions around the knee joint, resulting in decreased joint ROM. Additionally, the enhancement of joint ROM may be related to the shear forces generated by tissue flossing. The mechanical pressure applied by tissue flossing creates significant shear forces between different tissues, such as the epimysium, perimysium, and endomysium (Stecco et al., 2013). These shear forces facilitate the loosening of adhesive points and restore the lubricating action of hyaluronic acid, thereby contributing to an increase in joint ROM (Stecco et al., 2013).

EG exhibited better knee stability when compared to CG after the intervention. This suggests that acute tissue flossing can markedly improve proprioception and knee stability. Wu et al. (2022) reported no significant differences in single-leg standing (with eyes open or closed) or landing stability among participants immediately following an intervention using tissue flossing, a result that contrasts with the findings of this study. The main reason for the opposing results is that Wu et al. limited the maximum measurement time for single-leg standing time with eyes closed or open to 30 s, whereas the present study imposed no such restriction. The findings of the present study suggest two key factors contributing to the significant improvement in knee stability. First, the reduction in knee pain can directly enhance knee stability. One study indicates that pain triggers the release of inflammatory chemicals that sensitize nerve terminals, leading to abnormal firing of afferent nerve impulses, particularly from small-diameter pain-related nerves and large-diameter proprioceptive nerves (Prabhakar et al., 2023). This abnormal firing compromises knee stability. However, as pain decreases, this process is mitigated, thereby enhancing knee stability (Prabhakar et al., 2023). Second, the quadriceps muscles serve as dynamic stabilizer of the knee joint (Chang et al., 2021), and increased quadriceps muscle strength contributes to enhanced knee stability. Thus, the observed improvement in knee stability could be explained by the enhanced relative strength of the knee extensor muscles following the tissue flossing intervention.

In practice, the tissue flossing provides boxers with CKP a short-term, highly effective method for relieving knee pain, improving knee function, enhancing muscle strength and knee stability. Moreover, since this intervention does not require combination with other exercises, boxers with CKP can use it as an acute treatment before training or competitions.

## 5 Conclusion

Apart from ROM, applying tissue flossing for 3 min to boxers with CKP significantly alleviated knee pain and improved knee function scores, muscular strength and knee stability. The findings of this study can be applied to immediately relieve CKP symptoms in boxers and to offer an additional treatment option for athletes with CKP.

## 6 Limitation

In this study, we did not investigate the duration of the intervention effects or the trend of changes in various variables following immediate intervention. This can be explored further in future research.

## Data Availability

The datasets presented in this study can be found in online repositories. The names of the repository/repositories and accession number(s) can be found below: Jie, Chen; Wang, Dan (2024), “Acute Effects of Tissue Flossing on Boxers with Chronic Knee”, Mendeley Data, V1, doi: 10.17632/n46pws4vnb.1.
